# A Multi-Task Model for Pulmonary Nodule Segmentation and Classification

**DOI:** 10.3390/jimaging10090234

**Published:** 2024-09-20

**Authors:** Tiequn Tang, Rongfu Zhang

**Affiliations:** 1 School of Physics and Electronic Engineering, Fuyang Normal University, Fuyang 236037, China; 2 School of Optical-Electrical and Computer Engineering, University of Shanghai for Science and Technology, Shanghai 200093, China; zrf@usst.edu.cn; 3 Key Laboratory of Optical Technology and Instrument for Medicine, Ministry of Education, University of Shanghai for Science and Technology, Shanghai 200093, China

**Keywords:** lung nodule segmentation, lung nodule classification, multi-task network, prediction distillation, task correlation

## Abstract

In the computer-aided diagnosis of lung cancer, the automatic segmentation of pulmonary nodules and the classification of benign and malignant tumors are two fundamental tasks. However, deep learning models often overlook the potential benefits of task correlations in improving their respective performances, as they are typically designed for a single task only. Therefore, we propose a multi-task network (MT-Net) that integrates shared backbone architecture and a prediction distillation structure for the simultaneous segmentation and classification of pulmonary nodules. The model comprises a coarse segmentation subnetwork (Coarse Seg-net), a cooperative classification subnetwork (Class-net), and a cooperative segmentation subnetwork (Fine Seg-net). Coarse Seg-net and Fine Seg-net share identical structure, where Coarse Seg-net provides prior location information for the subsequent Fine Seg-net and Class-net, thereby boosting pulmonary nodule segmentation and classification performance. We quantitatively and qualitatively analyzed the performance of the model by using the public dataset LIDC-IDRI. Our results show that the model achieves a Dice similarity coefficient (*DI*) index of 83.2% for pulmonary nodule segmentation, as well as an accuracy (*ACC*) of 91.9% for benign and malignant pulmonary nodule classification, which is competitive with other state-of-the-art methods. The experimental results demonstrate that the performance of pulmonary nodule segmentation and classification can be improved by a unified model that leverages the potential correlation between tasks.

## 1. Introduction

Lung cancer is the most lethal cancer globally, and computed tomography (CT) has become one of the important means in reducing the mortality rates of advanced lung cancer due to its high resolution, high contrast and non-invasiveness. Radiologists observe and locate nodules in lung CT scans for lung cancer early screening and tracking lung tumor development, which not only requires significant professional skill, but also takes a long time and is prone to personal bias due to clinical experience. Therefore, computer-aided diagnosis (CAD) is urgently needed to help clinicians improve the accuracy and objectivity of diagnosis.

In recent years, more and more deep learning-based algorithms have been applied to the segmentation of pulmonary nodules and the classification of benign and malignant nodules. However, they are usually single-task studies that overlook the correlation between the segmentation of pulmonary nodules and the classification of benign and malignant nodules. The segmentation can provide certain location information for subsequent classification, which is beneficial for improving the accuracy of the benign and malignant classification of pulmonary nodules. Inspired by this, Yu et al. [1] utilized the segmentation results of pulmonary nodules to improve the classification accuracy. However, the classification results are too dependent on the segmentation performance, which increases the instability of the classification performance.

To address the above critical issues, we attempt to explore a novel multi-task network (MT-Net) for simultaneous lung tumor segmentation and classification of benign and malignant pulmonary nodules. Our network includes a coarse segmentation subnetwork (Coarse Seg-net), a cooperative segmentation subnetwork (Fine Seg-net) and a cooperative classification subnetwork (Class-net). The nodule masks generated by Coarse Seg-net are used to enhance the nodule discrimination ability of Class-net while providing additional location information for the accurate segmentation of pulmonary nodules by Fine Seg-net. As such, our MT-Net can improve both segmentation and classification performance. In addition, the application of rank loss in the segmentation subnetwork imposes additional constraints on the boundary pixels, which is conducive for solving the problem posed by fuzzy nodule boundaries. The competitive segmentation and classification performance of our method is verified by comparing it with other state-of-the-art deep learning algorithms on the publicly available dataset LIDC-IDRI [2]. In addition, the effectiveness of the idea of inter-task assistance in MT-Net is also validated by visualized ablation experiments. Overall, our contributions can be summarized as follows:

(1) We propose a multi-task network, MT-Net, for lung nodule segmentation and classification based on the inherent correlation between segmentation and classification tasks. MT-Net first generates coarse segmentation masks by Coarse Seg-net. Then, Fine Seg-net concatenates the coarse location information by the proposed Fine-layer for segmentation, and Class-net concatenates the coarse location information as its input for classification. The incorporation of prior location information facilitates the performance improvement of lung nodule segmentation and classification.

(2) A new hybrid loss is proposed in the segmentation subnetwork, which includes Dice loss and rank loss. In the segmentation task, in addition to considering the extreme imbalance of positive and negative samples in the CT patch, the importance of edge pixels for segmentation optimization is also considered. Rank loss poses extra constraints on the hard-to-recognize edge pixels, which effectively improves the accuracy of lung nodule segmentation.

(3) Experiments show that our method achieves a *DI* of 83.2% while achieving an *ACC* of 91.9%, which indicates that the overall performance of MT-Net is the most balanced and comprehensive and our method has competitive performance for lung nodule segmentation and benign–malignant classification compared to other state-of-the-art methods.

## 2. Related Works

With the development of deep learning technology, convolutional neural networks (CNNs) have been widely used in medical image analysis. There are many lung nodule segmentation methods based on deep learning [3,4,5,6,7,8]. UUnet first utilized the standard U-Net for coarse segmentation, and then applied attention U-Net with an attention gate (AG) as the subsequent fine segmentation model [3]. Based on the encoder–decoder structure, Wang et al. [5] added extra global attention units and edge losses to difficult-to-segment nodules. Zhu et al. [7] designed a high-resolution network with multi-scale progressive fusion in the encoder, and proposed a progressive decoding module (PDM) in the decoder. Similarly, there are lots of researchers who have focused on the classification of pulmonary nodules [9,10,11,12,13,14]. Lyu et al. [9] proposed a multi-level convolutional neural network (ML-CNN) composed of three CNN branches, in which the output of the pooling layer at the end of each CNN was flattened and concatenated to extract multi-scale features of lung nodules. Zhai et al. [12] constructed a new multi-task convolutional neural network (MT-CNN) framework to identify benign and malignant nodules on chest CT scans. MT-CNN learned 3D lung nodule features from nine 2D views from different angles of each nodule. Each 2D MT-CNN model consisted of two branches, one was the nodule classification branch (the main task) and the other was the image reconstruction branch (the auxiliary task). The motivation of the auxiliary task was to retain more microscopic information in the hierarchy of the CNN, which was conducive to the identification of malignant nodules. Zhang et al. [14] built an integrated learner for pulmonary nodule classification by fusing multiple deep CNN learners. Eight DCNN learners with different architectures were trained separately, so each nodule had eight predictions from eight major learners. The results showed that the prediction accuracy of the integrated learner (84.0% vs. 81.7%) was higher than that of the single CNN learner.

Although the performance of the above CNN-based methods has been further improved, they are all limited to a single segmentation or classification task and ignore the performance improvement that can be brought by the internal correlations between tasks. To solve this problem, Amyar et al. [15] adopted a multi-task deep learning model that consists of a generalized encoder, two decoders, and a multi-layer perceptron, where the generalized encoder can untangle features for all three tasks. It is a multi-task framework with a shared backbone structure. Liu et al. [16] applied a multi-task deep model with margin ranking loss (MTMR-Net) to the automatic pulmonary nodule analysis, in which the parallel layers of each task structure have information flow interactions. The model explicitly explored the correlation between pulmonary nodule classification and attribute score regression in a causal manner while producing results for different tasks. Both approaches explored the internal correlations between tasks; even so, the parallelism among tasks may result in insignificant performance improvement, which can be achieved by a prediction distillation structure.

The prediction results of one task in the distillation structure can assist another task to achieve higher-precision predictions. Wang et al. [17] proposed a cascade network architecture that can segment and classify ground glass nodules (GGNs) simultaneously, in which the segmentation model was used as a trainable pre-processing module to provide an attention weight map for classification guidance to the original CT data so as to achieve better nodule classification performance. Yu et al. [1] designed an algorithm based on a 3D ResU-Net segmentation network and a 3D ResNet50 classification network, which first segmented the nodules and then classified them as benign and malignant. Although these methods have achieved improvements, their performance in the second phase task was highly dependent on the results of the first phase task. Unlike these attempts, our method uses only the location information contained in the coarse segmentation mask as the auxiliary prior information for subsequent tasks, helping the segmentation and classification network to better identify nodules and avoiding the possible adverse effects of the poor performance of the first phase task on the final results.

## 3. Materials and Methods

### 3.1. Materials and Data Preprocessing

#### 3.1.1. Materials

The publicly available LIDC-IDRI dataset was used to evaluate the performance of our network. LIDC-IDRI is a dataset collected at the initiative of the National Cancer Institute in the United States, which contains a total of 1018 cases (the file type of the CT image data is Dicom) with corresponding diagnostic results. The images of each case were annotated by four experienced chest radiologists. Four physicians rated the malignancy of each nodule on a scale of 1 to 5, with higher scores indicating greater malignancy.

In the screening of the dataset, we first excluded nodules with a malignancy score of 3. We then identified nodules with a malignancy score of 1–2 as benign and with a score of 4–5 as malignant. Finally, we examined the nodules annotated by the four radiologists, and due to the variability among the four different radiologists, nodules that met the 2/3 consensus standard were adopted to generate ground truth. In addition, nodules with blurred identity documents (IDs) and that were smaller than 3 mm or larger than 25 mm were also excluded to avoid the interference of poor data for CNN model training. In the end, 1543 CT scans were selected, including 470 benign nodules and 1073 malignant nodules.

#### 3.1.2. Data Augmentation and Preprocessing

Firstly, the CT image and pixel-level label in LIDC-IDRI with a depth of 32 in RGBA mode were converted to images with a depth of 24 in RGB mode and a label with a depth of 8 in L mode. Then the nodule images were uniformly cropped into 64 × 64 CT patches. To avoid overfitting, we further augmented the number of images by 9 times in the pre-processing stage. Specifically, this involved actions such as randomly cropping based on the center of the image at a ratio of 50% to 100% of the original image size, zooming at an equal ratio of 110%, horizontally and vertically flipping, etc. Finally, images after data augmentation were resampled to 224 × 224. In this study, the ratio of 6:2:2 was adopted to divide the training, validation, and testing sets.

### 3.2. Method

#### 3.2.1. The Overall Structure of the Model

**Figure 1:**
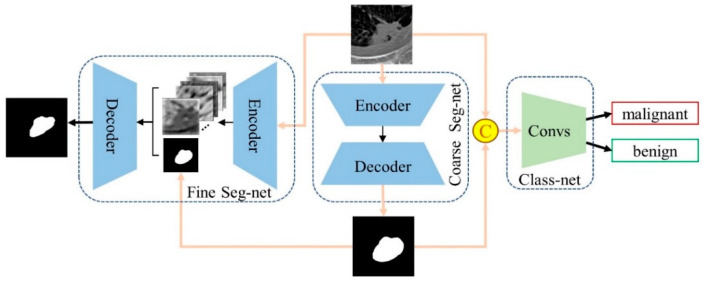
The pipeline of MT-Net, which consists of three subnetworks, Coarse Seg-net, Fine Seg-net, and Class-net.

Suppose that the segmentation training set and validation set with N1 and N2 images are represented as $I_{ST}={\left(X_{st},Y_{st}\right)}^{ST1}$ and $I_{SV}={\left(X_{sv},Y_{sv}\right)}^{SV1}$, respectively, where each image $X_{st}$ and $X_{sv}$ are labeled pixel-by-pixel, and each pixel belongs to a lung nodule (i.e., $Y_{sti}=1$) or background (i.e., $Y_{sti}=0$). Suppose that the classification training set and validation set with N1 and N2 images are represented as $I_{CT}={\left(X_{ct},Y_{ct}\right)}^{CT1}$ and $I_{CV}={\left(X_{cv},Y_{cv}\right)}^{CV1}$, respectively, where each image $X_{ct}$ and $X_{cv}$ are labeled at the image level (e.g., $Y_{ct},Y_{cv}\in \left\{l_{1},l_{2}\right\}$, where $l_{1}$ and $l_{2}$ denote the benign and malignant pulmonary nodules, respectively). The proposed MT-Net consists of three subnetworks, Coarse Seg-net, Fine Seg-net, and Class-net. Figure 1 shows the pipeline of this model. Different tasks can improve each other’s performance by transferring information to each other. First, Coarse Seg-net is trained on segmentation datasets $I_{ST}$ to generate the coarse mask of nodules from lung CT images. Then, we use the fine layer of Fine Seg-net to fuse the high-level features extracted from its encoder and the corresponding nodule localization map generated by Coarse Seg-net, which are then fed into the decoder of Fine Seg-net to obtain the fine segmentation of lung nodules. Finally, the original CT patches of pulmonary nodules on classification datasets $I_{CT}$ are concatenated with its corresponding nodule coarse masks generated by Coarse Seg-net and fed into Class-net to boost the performance of nodule benign–malignant classification.

#### 3.2.2. Coarse Seg-Net

**Figure 2:**
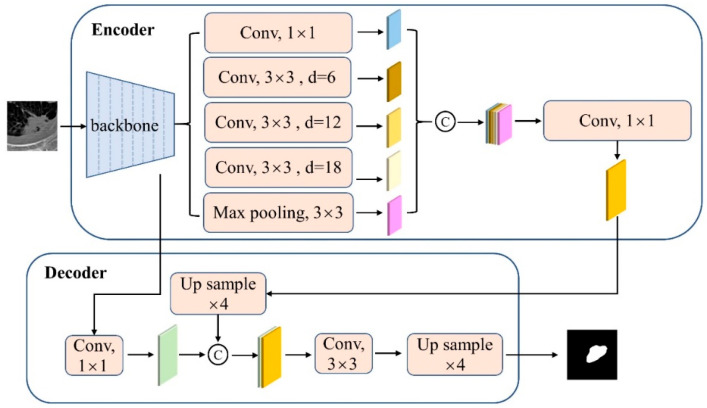
The structure of Coarse Seg-net, which is an improvement on Deeplabv3+ [18].

**Figure 3:**
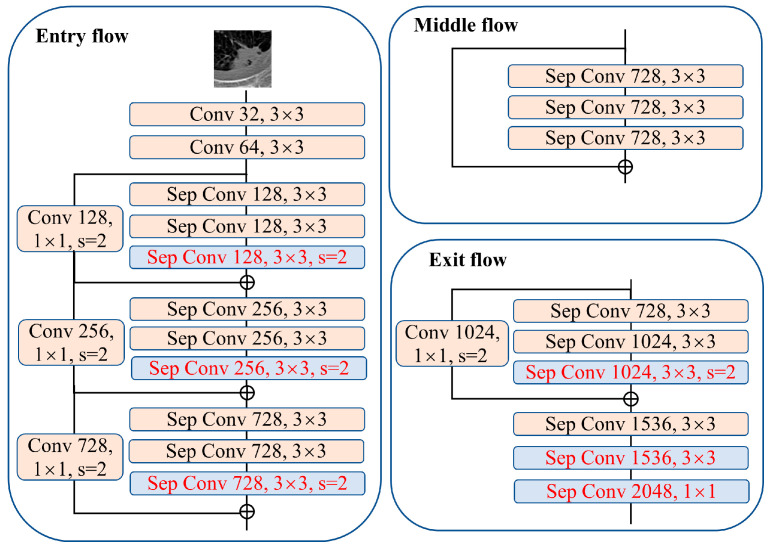
The structure of the backbone in the Coarse Seg-net encoder, which is a modified aligned Xception. The red font on the blue background is the modified layer.

Coarse Seg-net takes the segmentation training images as input with the aim of obtaining a coarse mask of the lung nodules, which provides the corresponding prior location information for the subsequent cooperative segmentation and classification subnetworks to enhance their localization and discrimination capabilities. Figure 2 shows the structure of Coarse Seg-net, which is an improvement on Deeplabv3+ [18] and pre-trained on the MS-COCO dataset [19]. Based on the understanding of the classical semantic segmentation network Deeplabv3+ and lung nodule segmentation, we employed a modified aligned Xception as the backbone in the encoder of Coarse Seg-net, as shown in Figure 3. The core idea is to replace the max pooling operation with a depthwise separable convolution with down-sampling (stride = 2) so that the resolution of the feature map can be enlarged by a dilated convolution. To better adapt to the setting of the pulmonary nodule segmentation task, a 1 × 1 convolution with output channel 1 and with a sigmoid activation function is used to replace the last 3 × 3 convolution of the network for predictions. The weights of the new layer are randomly initialized. In addition, a hybrid loss with rank loss is used to optimize Coarse Seg-net.

#### 3.2.3. Fine Seg-Net

**Figure 4:**
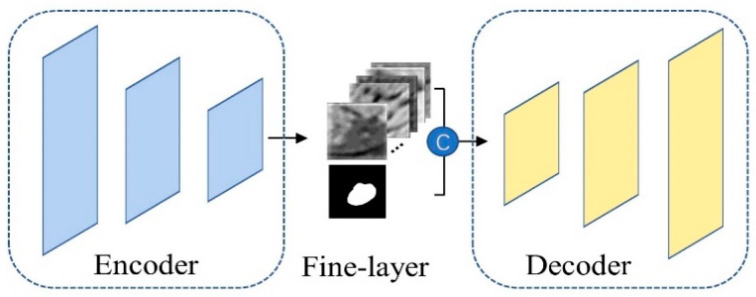
The architecture of Fine Seg-net, which employs the same architecture as the Coarse Seg-net. Fine-layer fuses the high-level semantic information from the encoder with the prior location information of the coarse segmentation mask.

Fine Seg-net takes the segmentation training images as input. As shown in Figure 4, it consists of an encoder, a decoder and a fine layer. The encoder and decoder of Fine Seg-net share the same structure and parameters with Coarse Seg-net but differ in that it introduces a new Fine-layer for receiving the coarse mask generated by Coarse Seg-net as a second input. The purpose of this new layer is to incorporate the prior location information from the coarse segmentation mask and the deepest high-level semantic features from the encoder to enhance the performance of Fine Seg-net in achieving accurate pulmonary nodule segmentation results. Specifically, Fine-layer first concatenates the feature maps generated by the encoder with the coarse segmentation mask. Then, a 1 × 1 convolutional layer followed by a batch normalization (BN) layer and a ReLU activation function is adopted for information fusion. Finally, the feature maps generated by Fine-layer are fed into the decoder for fine segmentation. The weights of Fine-layer are randomly initialized, and we also apply a hybrid loss with rank loss to optimize the Fine seg-net.

#### 3.2.4. Class-Net

**Figure 5:**
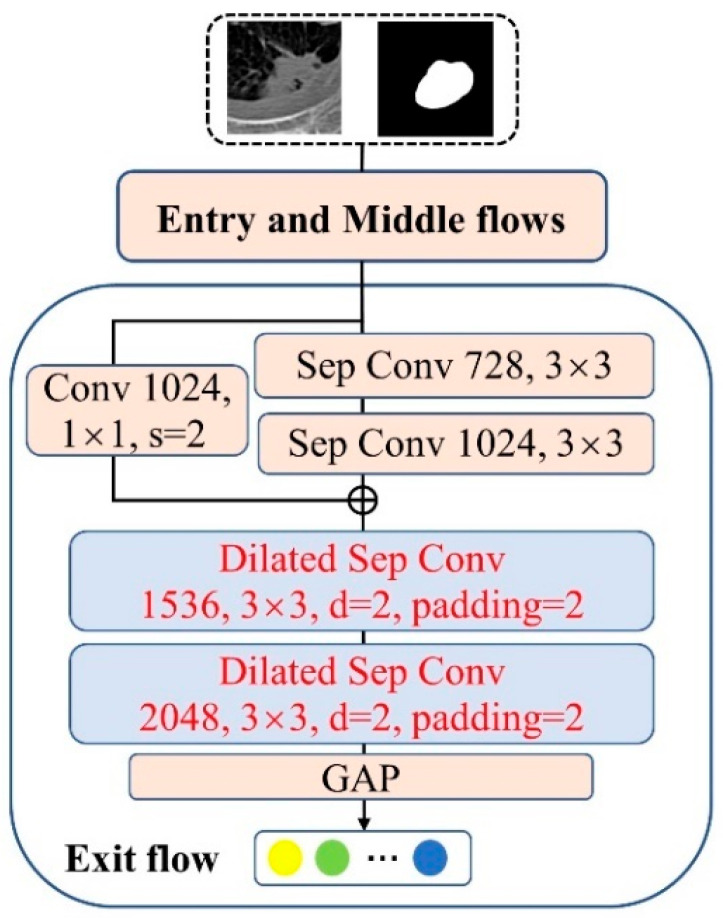
The structure of Class-net, where the last max pooling layer of its exit flow is replaced by two dilated separable convolutions with a dilated rate of 2 and padding = 2. The red font on the blue background is the modified layer.

For each segmented training image, it is fed into Class-net for classification training. Class-net utilizes coarse nodule masks generated by Coarse Seg-net to enhance its nodule location and discrimination ability. Specifically, the classification training images with image-level class labels and corresponding coarse masks are concatenated as inputs to Class-net. The weights of the coarse masks in the 4th channel are initialized by averaging the weights of the other three channels of the original RGB images. The structure of Class-net is shown in Figure 5, which is an improvement on the classification network Xception [20] and pre-trained on the ImageNet dataset [21]. The improvements are in the following two aspects: (1) the last max pooling layer in the exit flow of Xception is removed to preserve the resolution of the feature map and prevent the loss of small lung nodule details during progressive down-sampling. (2) To compensate for the reduced receptive field resulting from the removal of down-sampling in (1), the last two separable convolutions in the exit flow of Xception are replaced by a separable dilated convolution with a dilated rate of 2 and padding = 2. In addition, a binary cross-entropy loss is applied to optimize Class-net.

#### 3.2.5. Experiment Details

We selected Python3.6.2 as the programming language and PyTorch 1.4. to build the deep learning model. The algorithm was deployed on a device equipped with 2×Nvidia GTX1080 graphics card (made by Nvidia of Santa Clara, CA, USA) and 32GB of memory. We describe the implementation details as follows:

In the model training phase, we trained Coarse Seg-net and Fine seg-net using the LIDC-IDRI training set and validation set with pixel-level labels, and Class-net using the LIDC-IDRI training set with image-level labels. An Adam optimizer with a batch size of 16 and 32 was used to optimize the segmentation and classification networks, respectively. We set the initial learning rate to 1 × 10^−4^, the maximum number of epochs to 100, and set the hyper-parameters in the hybrid loss to λ=0.02,K=20, and margin=0.3. In the model testing phase, the trained MT-Net was directly applied to the LIDC-IDRI segmentation and classification testing set for lung nodule segmentation and classification.

#### 3.2.6. Loss Function

The overall framework consists of two tasks, segmentation and classification, and each task has a different loss function. Class-net adopts binary cross-entropy as the loss function of the classification network, while Coarse Seg-net and Fine Seg-net employ the hybrid loss.

(1) $$L_{\mathrm{seg}}=L_{\mathrm{Dice}}+\gamma L_{\mathrm{Rank}}$$

where γ is used to control the proportion of $L_{\mathrm{Rank}}$ in the hybrid loss.

Considering that the imbalance between the foreground and background in the segmentation image can have a negative impact on the performance, the segmentation subnetworks chose Dice loss [22], which has the ability to focus on the foreground region mining.

(2) $$L_{\mathrm{Dice}}=1-\frac{2\sum_{i=1}^{N}P_{i}G_{i}}{\sum_{i=1}^{N}(P_{i}+G_{i})+\varepsilon }$$

where N represents the total number of pixels, $P_{i}$ is the prediction probability of the i pixel belonging to the nodule, $G_{i}$ is the pixel-level label of the i pixel, and ε is the smoothing factor.

In general, pixels located at the edge of the nodule contribute more to the optimization of the segmentation results than those located in the internal area of the nodule and background. Due to the severe imbalance between edge and non-edge pixels in images, using Dice loss alone to train the segmentation network often cannot achieve good results. Inspired by the maximum-edge classification in [23,24], we therefore use rank loss for special supervision of pixels that are difficult to segment. Rank loss is an online rank scheme that dynamically selects edge pixels based on the prediction error. Specifically, the pixels of the nodule and background are ranked separately according to the error after the forward propagation of each batch. Rank loss is defined as follows:

(3) $$L_{\mathrm{Rank}}(P_{n},G_{n})=\frac{1}{K^{2}}\sum_{i=1}^{K}\sum_{j=1}^{K}\max\left\{0,H_{ni}^{0}(P_{n},G_{n})-H_{nj}^{1}(P_{n},G_{n})+margin\right\}$$

where K represents the number of pixels with the highest error in the manually selected foreground or background, that is, the difficult to segment pixels in this region. $H_{ni}^{0}$ and $H_{nj}^{1}$ denote the prediction probability values of the i difficult-to-segment pixel in the background and the j difficult-to-segment pixel in the nodule region of the n input image, respectively. margin is used to control the difference between the prediction probabilities of pixels in the foreground and background. In the training phase of the model, $H_{nj}^{1}(P_{n},G_{n})>H_{ni}^{0}(P_{n},G_{n})+margin$ is forced to control the segmentation network to pay more attention to the difficult-to-identify pixels.

## 4. Experimental Results

### 4.1. Evaluation Metrics

We select four general metrics to evaluate classification results, including accuracy (*ACC*), the area under the curve (AUC), sensitivity (*SEN*), and specificity (*SPE*), which is similar to other pulmonary nodule classifications [16,25,26]. They are defined as follows:

(4) $$ACC=\frac{TP+TN}{TP+TN+FP+FN}$$

(5) $$SEN=\frac{TP}{TP+FN}$$

(6) $$SPE=\frac{TN}{TN+FP}$$

where *TP*, *FP*, *TN*, and *FN* denote the number of true positives, false positives, true negatives, and false negatives, respectively.

The Dice similarity coefficient (*DI*) and Jaccard index (*JA*) are used to measure the overlap between two segmentation results. In addition, accuracy (*ACC*), recall and specificity (*SPE*) are also adopted to evaluate the segmentation performance of pulmonary nodules. These five metrics are also widely used in other segmentation works [27,28,29]. They are formulated as follows:

(7) $$DI=\frac{2|S\cap GT|}{\left|S|+|GT\right|}$$

(8) $$JA=\frac{\left|S\cap GT\right|}{\left|S|+|GT|-|S\cap GT\right|}$$

(9) $$ACC=\frac{I-|S\cup GT|+|S\cap GT|}{I}$$

(10) $$Recall=\frac{\left|S\cap GT\right|}{\left|GT\right|}$$

(11) $$SPE=\frac{I-|S\cup GT|}{I-|GT|}$$

where *S*, *GT*, and *I* represent the segmentation result, ground truth, and the original image, respectively.

### 4.2. Overall Performance Comparison with Other Multi-Task Networks

We compare our MT-Net with three state-of-the-art multi-task methods, as shown in Table 1. Wu et al. [30] proposed a multi-task method in which the segmentation encoder and the classification feature extractor share networks and parameters, and both segmentation and classification can update the parameters of the public network. Chen et al. [31] also adopted the same sharing strategy, but the difference is that it combined the features of segmentation prediction again in the feature extraction of classification to improve the classification performance. However, this approach of sharing pre-defined architectures limits the flexibility of the network for specific tasks. Yu et al. [1] designed the segmentation subnetwork and classification subnetwork as a serial structure, and the segmentation results were directly used as masks to highlight the nodules in the original image, which were then sent to the classification network for feature extraction. Nevertheless, this method relies heavily on the performance of the first subnetwork in the series and has some limitations in optimizing the performance of the second subnetwork. In contrast, the proposed MT-Net designs different subnetworks for different tasks, and the subsequent subnetworks only need the coarse location information generated by the coarse segmentation subnetwork, which avoids the impact of direct cropping on classification performance when the coarse segmentation is poor in the paper [1]. Specifically, our model transfers the nodule location information generated by the coarse segmentation subnetwork to the cooperative classification subnetwork to improve its nodule classification and location ability, and this nodule location information is also sent to the cooperative segmentation subnetwork to facilitate nodule segmentation. Table 1 shows that the average *DI* scores for segmentation, *ACC*, and AUC of the classification in our method are 2.7%, 4.6%, and 2.8% higher than Yu et al. [1]. Although the model designed by Wu et al. [30] outperforms the corresponding classification performance of our model in terms of the *ACC* score, its *DI* of segmentation is 9.3% lower than our model. Similarly, although the segmentation results of the multi-task network proposed by Chen et al. are higher than those of our method, the classification *ACC* metric decreases by 4.8%. In summary, MT-Net achieves competitive and the most balanced results on LIDC-IDRI datasets compared to the state-of-the-art methods.

### 4.3. Comparison of Segmentation Results

As shown in Table 2, we compare the proposed MT-Net with the recently published lung nodule segmentation methods. Our model achieves the best performance on the LIDC-IDRI dataset with *DI*, *JA*, *ACC*, recall, and *SPE* scores of 83.2%, 71.2%, 96.3%, 92.5%, and 97.7%, respectively. In particular, our model increases the average *DI* score from 83.0% to 83.2% compared to the second-best approach of Ni et al. [32]. This indicates that our model achieves better performance and higher accuracy in the task at hand.

### 4.4. Comparison of Benign and Malignant Classification Results

In our comparison with other recently proposed classification methods, as shown in Table 3, our proposed MT-Net outperforms almost all other methods. On the LIDC-IDRI testing sets, MT-Net achieves the highest *ACC* of 91.9% and surpasses the second-ranked model in terms of *ACC* by 0.8%. This demonstrates that MT-Net exhibits superior performance and achieves higher accuracy in the classification of a given dataset.

### 4.5. Visual Results

**Figure 6:**
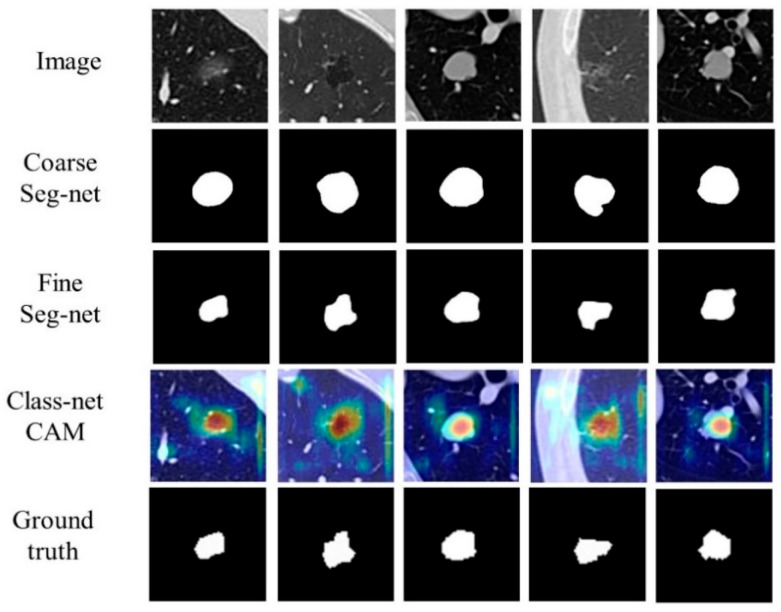
Sample visualization of prediction for each segmentation subnetwork and classification subnetwork.

In Figure 6, we present the visualizations of the results obtained from our method, including the coarse segmentation, fine segmentation, and classified class activation mapping (CAM) [37]. It is evident that the nodule coarse segmentation information generated by Coarse Seg-net plays a crucial role in guiding Fine Seg-net to achieve more precise segmentation results. Additionally, this coarse segmentation information helps Class-net to produce more accurate nodule localization during classification. Overall, the combination of these tasks in our approach allows for improved segmentation and classification, leading to enhanced performance in the given task.

### 4.6. Ablation Experiment

**Figure 7:**
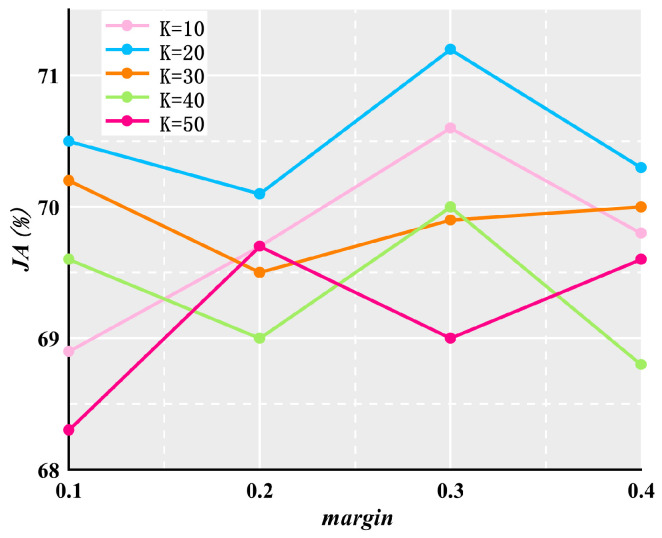
Effects of different settings of K and *margin* values on *JA*.

In rank loss, the hyper-parameters K and margin denote the number of selected difficult-to-segment pixels and the constraint between the predicted values of the background and foreground difficult-to-segment pixels, respectively. To explore the effect of their settings on the segmentation, K is set to 10, 20, 30, 40, and 50, and margin is set to 0.1, 0.2, 0.3, and 0.4, respectively. Figure 7 plots the *JA* values corresponding to different K values and margin values. It can be observed that the highest *JA* value is attained when K = 20 and margin = 0.3. Consequently, we adopt *K* = 20 and margin = 0.3 as the selected hyper-parameter values.

**Figure 8:**
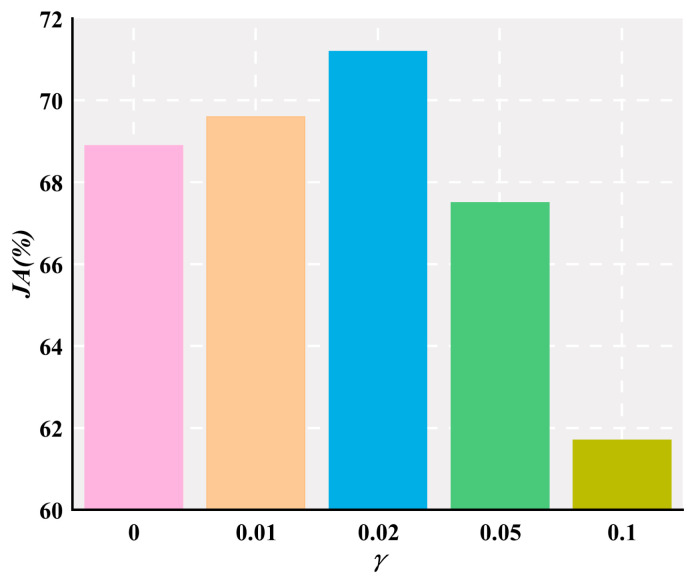
Effects of different settings of γ values on *JA*.

In addition, the weighting factor γ controls the proportion of rank loss contributing to the hybrid loss in the segmentation task. In order to investigate the influence of different settings of this parameter on the experiment, the nodule segmentation is repeated with K fixed at 20 and margin fixed at 0.3 by setting γ to 0, 0.01, 0.02, 0.05, and 0.1. Figure 8 shows JA values corresponding to different γ on the LIDC-IDRI testing set. It is clear that the proposed model achieves the highest *JA* when γ is set to 0.02. Thus, γ = 0.02 is used as the default weighting factor for rank loss.

The coarse masks of nodules predicted by Coarse Seg-net are used to boost the classification ability of Class-net. To assess the effectiveness of this concatenation strategy, we compare the discrimination ability with and without Coarse seg-net on the LIDC-IDRI testing set in Table 4. It can be seen that combined with the auxiliary role of Coarse Seg-net, the model achieves higher accuracy in nodule classification, with the AUC increasing from 90.6% to 93.5%. This is mainly attributed to the fact that the predicted nodule location maps allow Class-net to focus more on the nodule region rather than the background on the CT image.

To evaluate the impact of introducing the nodule location maps on the segmentation performance, the segmentation performance with and without the introduction of prior location information is compared on the LIDC-IDRI testing set in Table 5. It is observed that Fine seg-net has better segmentation performance (i.e., *DI* increased from 80.1% to 83.2%) when guided by Coarse seg-net.

## 5. Discussion

With the development of CT imaging technology and deep learning technology, CAD is more and more widely used in the medical fields. A CAD system of lung cancer includes two crucial tasks, the segmentation of lung nodules and the classification of benign and malignant tumors. However, the difficulty of accurate segmentation and classification of pulmonary nodules is increased due to the following reasons: (1) there is significant intra-class heterogeneity between different nodules; (2) there is high inter-class similarity between nodules and normal tissues; and (3) pulmonary nodules often have complex backgrounds and fuzzy boundaries. Considering that single-task studies often ignore the correlation between different tasks, we designed a multi-task network (MT-Net) to perform lung nodule segmentation and benign–malignant tumor classification simultaneously.

Multi-task frameworks apply different modules to handle different tasks. Our framework implements the segmentation process of lung nodules from coarse to fine by sharing the same basic modules and parameters, and transmits the location of the coarse segmentation of lung nodules as auxiliary prior information to the pulmonary nodule classification task by a distillation structure, which avoids the bias that may be caused by the decisive role of the first phase results. In addition, rank loss is used to impose extra constraints on edge pixels that play a key role in segmentation tasks. Experiments show that our method has both a high *DI* value for segmentation and a high *ACC* value for classification.

While our multi-task approach performs well compared to other state-of-the-art multi-task methods, it lacks feedback or the facilitation of information from classification to segmentation. In the future, we will explore the promotion of tasks to each other, maximize the mutual enhancement between tasks, and even incorporate a detection task to maximize the effectiveness of the multi-task model. In addition to improving the model to serve as a feature extractor, the appropriate parameter selection of the deployed pre-trained model may also have better performance [38,39,40]. This inspires us to rethink parametric fine-tuning technologies, and we will focus on parametric fine-tuning in the future. In addition, we also note the role of microribonucleic acid (miRNA) expression in the staging diagnosis and treatment of cancers such as lung cancer [41,42,43,44], as well as the role of endoscopic ultrasound (EUS) in the diagnosis and treatment of tumors, which allows for a more detailed classification and complete staging of lung nodules in a single session, with complementary effects [45,46,47]. In the future, we may integrate miRNA information or EUS images to design a multi-modality deep learning model for boosting the accuracy of the auxiliary diagnosis of lung cancer.

## 6. Conclusions

In this paper, we propose a multi-task model for lung nodule segmentation and benign–malignant classification based on the idea that the correlations existing between tasks can boost performance. Our approach is to introduce the coarse localization information of the coarse segmentation mask into the cooperative segmentation subnetwork (Fine Seg-net) and cooperative classification subnetwork (Class-net) to improve the performance of lung nodule segmentation and classification. Experimental results show that MT-Net achieves a *DI* of 83.2% for segmentation and an *ACC* of 91.9% for benign and malignant classification, which are comparable to the overall segmentation and classification performance of the multi-task model. In terms of single-task performance, the segmentation result of MT-Net is 0.2% higher than the second-ranked model from Ni et al. [32] in *DI* score, and 0.8% higher than the second-ranked model from Huang et al. [36] in terms of the *ACC* index for the classification of MT-Net. In addition, the visualized segmentation results and CAM for classification indicate that the coarse segmentation information of the nodules helps to guide the cooperative segmentation subnetwork to obtain better segmentation results. It also facilitates the cooperative classification subnetwork to produce more accurate nodule localization.

## Figures and Tables

**Table 1:** Comparison of pulmonary nodule segmentation and classification performance with other three multi-task methods.

	Tasks	Segmentation					Classification			
Methods		*DI* (%)	*JA* (%)	*ACC* (%)	Recall (%)	*SPE* (%)	*ACC* (%)	AUC (%)	*SEN* (%)	*SPE* (%)
[30]		73.9	-	-	-	-	97.6	-	-	-
[31]		86.4	77.1	-	-	-	87.1	-	-	-
[1]		80.5	-	-	80.5	-	87.3	90.7	-	-
Ours		83.2	71.2	96.3	92.5	97.7	91.9	93.5	81.4	95.0

“-” indicates that there is no corresponding parameter in the paper.

**Table 2:** Comparison with other latest segmentation methods.

Methods	Year	*DI* (%)	*JA* (%)	*ACC* (%)	Recall (%)	*SPE* (%)
[33]	2018	78.0	64.0	-	86.0	-
[4]	2019	81.6	68.9	-	87.3	-
[34]	2020	82.7	70.5	-	89.4	-
[6]	2021	82.5	70.2	-	82.3	-
[32]	2022	83.0	71.0	-	-	-
Ours		83.2	71.2	96.3	92.5	97.7

“-” indicates that there is no corresponding parameter in the paper.

**Table 3:** Comparison with other latest classification methods.

Methods	Year	*ACC* (%)	AUC (%)	*SEN* (%)	*SPE* (%)
[25]	2018	83.5	91.2	80.5	86.0
[35]	2019	84.2	85.6	70.5	88.9
[26]	2021	84.3	91.6	84.5	83.8
[36]	2022	91.1	95.8	-	-
Ours		91.9	93.5	81.4	95.0

“-” indicates that there is no corresponding parameter in the paper.

**Table 4:** Pulmonary nodule classification performance with and without Coarse seg-net.

Coarse Seg-Net	Class-Net	*ACC* (%)	AUC (%)	*SEN* (%)	*SPE* (%)
✕	√	84.2	90.6	80.1	92.5
√	√	91.9	93.5	81.4	95.0

**Table 5:** Pulmonary nodule segmentation performance with and without Coarse seg-net.

Coarse Seg-Net	Fine Seg-Net	*DI* (%)	*JA* (%)	*ACC* (%)	Recall (%)	*SPE* (%)
√	✕	80.1	66.8	94.9	93.4	98.2
√	√	83.2	71.2	96.3	92.5	97.7

## Data Availability

The LIDC-IDRI dataset presented in this study is derived from public domain resources. The images obtained from reference [2] are all available at https://www.cancerimagingarchive.net/collection/lidc-idri/ (accessed on 17 September 2024).
